# Lived experience perspectives on a definition of eating disorder recovery in a sample of predominantly white women: a mixed method study

**DOI:** 10.1186/s40337-022-00670-2

**Published:** 2022-10-13

**Authors:** Therese E. Kenny, Kathryn Trottier, Stephen P. Lewis

**Affiliations:** 1 Department of Psychology, University of Guelph, 50 Stone Road E., Guelph, ON N1G 2W1 Canada; 2 University Health Network, 200 Elizabeth Street, Toronto, ON M5G 2C4 Canada; 3 Department of Psychiatry, University of Toronto, Toronto, ON Canada

**Keywords:** Eating disorder, Recovery, Lived experience, Qualitative, Thematic analysis

## Abstract

**Background:**

There has recently been a push for recovery-focused research in the eating disorder (ED) field, starting with a consensus definition of recovery. One definition, in particular, proposed by Bardone-Cone et al. [21] has received considerable attention given its transdiagnostic nature and validation studies. However, no studies to date have elicited lived experience views of this definition. The goal of the current study was to examine perspectives on this definition of recovery from individuals with a past or present ED and to determine whether participant agreement with the model differed based on diagnostic history or current symptom severity.

**Methods:**

Sixty-two individuals (95.2% women; 91.9% White/European) participated in a 1–2 h interview aimed at capturing their perspectives on ED recovery. Transcripts were analyzed using qualitative content analysis and codebook thematic analysis to examine agreement with and thoughts on Bardone-Cone’s definition of recovery, respectively. Chi-squared tests of independence and binary logistic regression were computed to determine whether agreement with the definition differed across diagnostic history and self-reported symptoms.

**Results:**

Although some participants indicated acceptance of the definition, the majority expressed concerns related to its categorical nature, proposed criteria, feasibility, language, and applications. There were no differences in acceptance based on diagnostic history or current symptom severity.

**Conclusion:**

A single definition of recovery does not seem to fit individuals’ lived ED experience. Future research may benefit from distinguishing between recovery as an individually-defined phenomenon and related constructs such as remission (i.e., loss of diagnosis or absence of clinical symptoms). A more comprehensive multi-faceted, and person-centered model of recovery may have merit in clinical settings.

## Plain English Summary

Eating disorder recovery has been defined as reaching a specific body weight, while also refraining from eating disorder behaviours and reporting lessened eating disorder thoughts. Although this seems reasonable, no studies have asked people who have or have had eating disorders if this fits with their lived experience. We presented participants with a specific definition of eating disorder recovery which includes weight, behaviour, and thought criteria and asked them about their perspectives. Though some people felt this definition was sufficient, the majority expressed concerns. These ranged from concerns about the specific criteria (e.g., using body weight is outdated and not informative) to the applications of the definition (e.g., such specific criteria may limit some people from receiving services). These findings point toward the inclusion of lived experience voices in eating disorder recovery definitions.

## Background

Eating disorders (EDs) are serious mental illnesses characterized by unhelpful and distressing eating patterns (e.g., food restriction, binge eating, compensatory behaviours) and body image disturbance [1]. Findings from longitudinal studies suggest that even with treatment, the course of EDs is often persistent [2–5], and of people whose symptoms remit, many relapse (e.g., [6, 7]). Moreover, EDs are associated with significant mental (e.g., [8–10]) and physical [11] health consequences and are considered to be among the deadliest of mental illnesses [12–14]. This poor prognosis notwithstanding, there is a subset of individuals with EDs who make significant improvements and achieve what is typically referred to as “recovery” in clinical and research settings (e.g., [13]). Understanding factors that promote recovery attainment may, therefore, have implications in improving treatment efficacy and client outcomes.

There has recently been a push for recovery-focused research in the ED field, starting with a consensus recovery definition that considers recovery as endpoint that can be measured according to specific and well-defined criteria. Developing such a definition would ostensibly allow researchers to compare recovery rates across studies, examine predictors and barriers to recovery, and explore neurobiological underpinnings of recovery [15]. Clinically, such a definition may aide treatment providers in treatment planning, such as in making decisions about changing levels of care or terminating services [16]. Despite these well-intentioned motivations for a singular recovery definition, no such consensus exists [15].

Indeed, numerous definitions of ED recovery have been proposed, varying in symptom criteria and duration (e.g., [15, 17, 18]), which has resulted in recovery rates ranging from 8 to 88% depending on the definition used [13, 17–20]. To address this, Ackard et al. [19] compared seven different definitions and recommended using a definition of recovery put forth by Bardone-Cone et al. [21]. Since its publication, this model has become popular in the ED field, with over 300 citations on Google Scholar as of March 2022; it has also been highlighted in plenaries on recovery at the annual meeting of the Academy for Eating Disorders [22] and featured in the International Journal of Eating Disorders’ recent special issue on recovery (e.g., [23]).

This set of recovery criteria (hereafter referred to as the Bardone-Cone’s defitinition of recovery) proposes that there are two ‘types’ of recovery: full and partial. Individuals are characterized as being in full recovery when they: (1) have a body mass index (BMI) over 18.5; (2) have not engaged in binge eating, purging, or fasting in the past three months; and (3) report a score on the Eating Disorder Examination—Questionnaire (EDE-Q) less than one standard deviation above community norms, indicating ‘normalized’ ED psychopathology. Individuals considered to be in partial recovery meet the first two criteria but report an EDE-Q score greater than one standard deviation above community norms [21].

In the original validation study, individuals in the full recovery group reported ED psychopathology scores and co-occurring Axis-I diagnoses similar to individuals who had never had an ED, whereas individuals in partial recovery reported scores that were not significantly different from individuals with a current ED [21]. Subsequent studies have reported a similar pattern of results on measures of perfectionism, self-concept, coping, negative affect, negative urgency, eating expectancies, objectified body consciousness, motivation for exercise, and body appreciation and intuitive eating [23–31]. There is also preliminary evidence that this definition is valid among men [32] and predictive of sustained ‘full recovery’ status as defined by the Bardone-Cone definition [33]. It is important to note however, that all of these studies except for Bardone-Cone et al. [32] were conducted in the same sample of women recruited from a child and adolescent treatment program, calling into question the generalizability of this model.

Moreover, Bardone-Cone’s defitinition of recovery is aligned with the medical model of recovery which positions recovery as the absence of symptoms and return to pre-morbid functioning (see [34] for discussion). This is contrasted by the recovery model which emphasizes the personal experience of recovery. The recovery model gained prominence in the 1980’s and 1990’s, as a response to the generally poor prognosis for individuals with mental illness, primarily schizophrenia [35]. At this time, individuals with schizophrenia began sharing stories of recovery that were not predicated on symptom abatement (e.g., [36]) which led to a redefinition of ‘recovery’ in the field. For example, Anthony [37] described recovery as ‘a way of living a satisfying, hopeful, and contributing life even with the limitations caused by illness’ (pp. 527). From this perspective, and consistent with mental health policy in many countries (e.g., [35]), clinical and medical knowledge is important but individuals with lived experience are considered the experts in their own recovery. There have been arguments that this framework may hold values in the context of eating disorders, specifically anorexia nervosa [34].

In line with this, recent calls to action have highlighted the importance of integrating lived experience perspectives as these individuals are arguably experts in the experience of EDs and recovery [16, 38]. By discounting lived experience views, we run the risk of missing key considerations in ED recovery, assuming that one size fits all, and invalidating individuals whose experiences are not represented (typically those in already underrepresented in the ED field and marginalized groups). The integration of such views with clinical/research expertise may allow for a more comprehensive understanding of recovery which considers multiple stakeholders in the development of important constructs. This notwithstanding, no studies have examined how the Bardone-Cone recovery criteria fit with the perspectives of individuals with lived experience.

Given that qualitative studies of ED recovery highlight aspects beyond ED pathology (e.g., [39–41]), it is likely that individuals with lived experience will express concerns about or not agree with the Bardone-Cone definition of recovery. Recently, McDonald et al. [42] examined ED therapist and service user perspectives on a model of recovery from anorexia nervosa. In this study, participants were presented with a peer-reviewed article describing proposed criteria for recovery [43] and asked questions about their views on the definition. Findings indicated that service users saw the importance of having a universally recognized definition of recovery but had concerns with the definition itself. Specifically, service users felt that aspects of recovery such as coping and quality of life were missing, and some had concerns about the focus on BMI in the definition. Finally, all service users in the study had concerns that the proposed duration of one year without symptoms was too short and oversimplified the recovery process [42]. Thus, it follows that individuals with lived experience may have similar concerns about the Bardone-Cone definition of recovery.

Drawing from this, and recent calls to integrate lived experience into ED research broadly [39] and existing ED recovery frameworks more specifically [40], the goal of the current study was to examine lived experience perspectives on the full and partial recovery criteria proposed by Bardone-Cone et al. [21] using a mixed-method approach. In considering how participants with lived experience viewed these criteria, we also looked at potential differences in their agreement with the model across their diagnostic and symptom history, as well as their recovery status just prior to the interview, using quantitative analyses. It was hypothesized that individuals who had only experienced a diagnosis of anorexia nervosa would be more likely to endorse the model given that the three components map better onto the diagnostic criteria of anorexia nervosa compared to other ED diagnoses [1], and that individuals with no current ED symptoms and lower ED psychopathology would be more likely to express full agreement because the criteria (e.g., abstinence) may seem more achievable to them. Similarly, we hypothesized that individuals meeting full recovery criteria at the time of the screening questionnaire would be more likely to endorse the Bardone-Cone definition of recovery, as they had achieved these criteria.

## Method

### Participants

Three hundred and seventy-eight individuals initially completed the screening questionnaire for this study. Of these, 195 provided an email address to be contacted for a follow-up video interview. Two of these individuals were not eligible to participate because of a personal connection with the lead interviewer and a lack of past or present ED diagnosis. Thus, 193 invitations were sent out to participate in the interview portion of the study. Sixty-five interviews were scheduled and 63 took place. A third interview was excluded from the analyses because the participant became upset during the interview and was not able to answer the necessary interview questions. Thus, data from 62 interviews were included in the analyses.

Participants reported a mean age of 30.34 years (*SD* = 9.988; range 18–66). The majority identified as women (95.2%) and reported White/European ancestry (91.9%). With respect to clinical characteristics, 59 participants (95.2%) reported receiving a formal diagnosis from a medical or mental health professional, with the majority reporting a diagnosis of anorexia nervosa (74.2%). Fewer participants reported having been diagnosed with bulimia nervosa (17.7%), other specified feeding or eating disorder (OSFED formerly EDNOS; 14.5%), and binge-eating disorder (3.2%). Most participants had received treatment in the past (96.8%), with a minority still receiving services (37.1%), mostly in the form of outpatient treatment (24.4%). Most of the sample also reported having been diagnosed with another mental illness (85.5%) and/or experiencing undiagnosed mental health challenges (40.3%). Clinical and demographic data are presented in Tables 1, 2 and 3.

**Table 1:** Demographic characteristics of the sample

Demographic characteristics	Frequency (%)
*Gender*	
Woman	59 (95.2)
Man	0 (0)
Not listed	3 (4.8%)
*Ethnicity*	
Black/African/Caribbean	2 (3.2)
Indigenous	2 (3.2)
Latin American	2 (3.2)
South Asian	4 (6.5)
Southeast Asian	2 (3.2)
West Asian	0 (0)
White/European	57 (91.9)
Not listed	2 (3.2)
*Sexual orientation*	
Heterosexual	50 (80.6)
Lesbian	1 (1.6)
Bisexual	5 (8.1)
Queer	5 (8.1)
Not listed	1 (1.6)
*Relationship status*	
Married	11 (17.7)
Cohabitating	11 (17.7)
Single, never married	37 (59.7)
Not listed	3 (4.8)
*Education*	
Completed high school	3 (4.8)
Some College/University	10 (16.1)
Completed College/University	17 (27.4)
Some graduate education	9 (14.5)
Completed graduate education	11 (17.7)
Completed professional program	12 (19.4)
*Employment status*	
Student	20 (32.3)
Full-time	22 (35.5)
Part-time	11 (17.7)
Unemployed	6 (9.7)
Medical leave	2 (3.2)

Percentages for ethnicity do not add up to 100% because participants could endorse more than one ethnic identity

**Table 2:** Eating disorder history

Eating disorder	Frequency (%)
*Diagnosed*	59 (95.2%)
Anorexia nervosa	46 (74.2)
Binge-eating disorder	2 (3.2)
Bulimia nervosa	11 (17.7)
Other specified feeding or eating disorder (OSFED)—formerly eating disorder not otherwise specified (EDNOS)	9 (14.5)
*Past treatment*	60 (96.8)
Inpatient/residential	32 (51.6)
Medical hospitalization	28 (45.2)
Outpatient	43 (68.3)
Day hospital	24 (38.7)
Partial inpatient	12 (19.4)
Private practice	28 (45.2)
*Current treatment*	23 (37.1)
Inpatient/residential	1 (1.6)
Medical hospitalization	1 (1.6)
Outpatient	15 (24.2)
Day hospital	0 (0)
Partial inpatient	0 (0)
Private practice	11 (17.7%)

Percentages for eating disorder diagnoses do not add up to 95.2% because participants endorsed more than one diagnosis

**Table 3:** Mental health history

Other mental health challenges	Frequency (%)
Diagnosed	53 (85.5)
Anxiety disorder	40 (64.5)
Mood disorder	41 (66.1)
Obsessive–compulsive disorder	9 (14.5)
Post-traumatic stress disorder	8 (12.9)
Personality disorder	9 (14.5)
Neurodevelopmental disorder	2 (3.2)
Substance use disorder	1 (1.6)
Schizophrenia spectrum	1 (1.6)
Undiagnosed	25 (40.3)
Anxiety	11 (17.7)
Low mood	9 (14.5)
Obsessive–compulsive symptoms	3 (4.8)
Borderline personality disorder symptoms	2 (3.2)
Trichotillomania/excoriation	3 (4.8)
Trauma	2 (3.2)
Neurodevelopmental difficulties	1 (1.6)
Dissociation	1 (1.6)
Past mental health treatment	53 (85.5%)
Current mental health treatment	38 (61.3%)

With respect to recovery status, the majority of our sample did not meet the criteria for full or partial recovery (n = 44, 71.0%). The remainder of the sample was equally split between full (n = 9, 14.5%) and partial (n = 9, 14.5%) recovery. Of note, there were a number of participants who met the cognitive criterion but not the weight and/or behavioural criteria and thus, were categorized as not meeting any recovery criteria despite having ‘normalized’ ED psychopathology.

### Measures

#### Demographics

Participants provided information across a variety of demographic (e.g., age, ethnicity, sexual orientation) and clinical (e.g., diagnosis and treatment history) variables on the screening questionnaire.

#### Eating disorders examination lifetime interview

The Eating Disorder Examination (EDE) [44] is a well-validated (e.g., [45]) semi-structured, investigator-based interview that assesses the frequency and severity of ED symptoms. The current study employed a modified lifetime interview [5] to confirm that participants met criteria for an ED at some point in their life. Adjustments were made to account for the transition from DSM-IV-TR to DSM-5 (e.g., change in frequency criterion for bulimia nervosa and binge-eating disorder).

#### Eating Disorders Examination—Questionnaire

The EDE—Questionnaire (EDE-Q) [46, 47] is a self-report measure of ED psychopathology, derived from the EDE. While we used the full EDE-Q to classify participants’ recovery status (consistent with the original definition), studies have demonstrated that a brief 7-item version shows stronger psychometric properties among individuals with and without EDs [48]. These subscales were therefore, computed for descriptive purposes. In the current study, the three EDE-Q Short-form subscales (i.e., dietary restraint, overvaluation of weight and shape, body dissatisfaction) and global scale showed good to excellent internal consistency (Cronbach’s alpha = 0.72–0.96).

Participants reported an average EDE-Q Global score of 2.90 (*SD* = 1.52), which is below the clinical cut-off of 4 [46]. Subscale scores were variable with participants reporting the highest scores for overvaluation of weight and shape (*M* = 3.40, *SD* = 1.97) and body dissatisfaction (*M* = 3.59; *SD* = 1.93) and lowest scores for dietary restraint (*M* = 1.72; *SD* = 1.62). On the lifetime Eating Disorder Examination (EDE) interview, 28 individuals (45.2%) reported a history of behavioural ED symptoms including fasting, binge eating, purging and other compensatory behaviours in the four weeks leading up to the interview.

#### Open-ended questions about the Bardone-Cone definition

Participants responded to a series of open-ended questions about their recovery experience. A subset of those questions^1^ were analyzed to ascertain individuals’ perspectives on the recovery criteria proposed by Bardone-Cone et al. [21]. Participants were presented with a lay-summary of the Bardone-Cone definitions of full and partial recovery (Table 4). If participants had questions about the criteria (e.g., ‘How do they measure thoughts?’), these were answered. Similarly, if participants’ responses indicated a misunderstanding of the definition (e.g., ‘It’s unrealistic for everyone to have a BMI of 18.5’), these were corrected. They were then asked about their thoughts on each of the definitions (e.g., *What are your thoughts on full recovery?*). This question was left intentionally vague so as to not influence responses. Participants were also asked how appropriate they felt each definition to be. The specific wording of these questions is presented in Table 4.

**Table 4:** Questions asked in the interview and included in the current analysis

Qualitative questions
*Some people talk about full and partial recovery. In research, full recovery is if your body weight is in the normal range, you have not engaged in ANY eating disorder behaviours (e.g., binge eating, purging) in the past three months, and you also have minimal ED thoughts (e.g., feeling fat, guilty about eating)*
1. What are your thoughts on full recovery? How appropriate is it to talk about full recovery?
2. Is it possible to be fully recovered from an eating disorder?
*Partial recovery is if your body weight is in the normal range and you have not engaged in ANY eating disorder behaviours (e.g., binge eating, purging) in the past three months, but you still have ED thoughts*
3. What are your thoughts on partial recovery? How appropriate is it to talk about partial recovery?
*These criteria talk about weight, behaviours, and thoughts*
4. Is there anything missing from these criteria?

### Procedure

Before data collection, the current study received ethics clearance from the University of Guelph research ethics board (REB #19-04-014). Prospective participants were recruited via online advertisements distributed via the researchers, their colleagues, and ED organizations over social media. To recruit individuals with a diverse range of experiences (i.e., individuals who do and do not identify with the term ‘recovery’), advertisements requested participation from individuals who had previously experienced an ED and had made improvements in their eating, with no specifications of what improvements may entail. Interested individuals were directed to an online screening questionnaire delivered via Qualtrics XM, Version October 2019, (Qualtrics, Provo, UT, USA). As part of this questionnaire, participants provided demographic and clinical information, completed standardized questionnaires (e.g., EDE-Q), and responded to open-ended questions. At the end of the survey, participants were invited to provide an email address if they wished to be contacted for a follow-up interview.

Individuals who provided an email address were invited to take part in a 1–2 h virtual semi-structured interview via WebEx, a secure video conferencing platform. Participants taking part in this interview were informed about the purpose of the study and asked to give verbal informed consent. They were then asked a series of questions about their recovery experience. At the end of the interview, we checked-in to confirm each participant’s willingness to have their data included in the study and provided information about potential resources such as ED-specific helplines. Interviews were transcribed by a trained undergraduate volunteer and reviewed (i.e., the transcript was reviewed while listening to the recorded interview) by the first author.

### Reflexivity statement

We acknowledge that the identities held by the research team will impact data collection, analysis, and interpretation. Thus, before describing the analytic approach, we provide a reflexivity statement. We are all white cis researchers and/or clinicians currently employed by academic institutions which invariably situates us in positions of power. It is possible that this power differential may have impacted who was willing to participate and what participants were willing to share during the interview. Interviews were conducted by TEK, a PhD student, and efforts were made to create a comfortable and safe space for sharing information. At the time of the interviews, TEK was affiliated with two regional ED advocacy groups but not providing direct services to clients. We hoped that this would allow participants to talk freely about treatment experiences with someone not directly tied to these services. The analysis was led by the first author (TEK) who identifies as someone with lived experience with/of an eating disorder (ED). At the time of data collection, TEK identified as someone ‘in recovery;’ however, her view on recovery more broadly has shifted over the course and in parallel with this research. She now identifies as someone who had an ED and appreciates that others may have differing relationships with the term ‘recovery.’ Throughout the analytic process, TEK kept a reflective journal and consulted with SPL to process elements of the dataset that felt relatable, close to, or distant from TEK’s personal experience. SPL is a researcher who has published and advocated for lived experience and person-centered approaches in the context of self-injury and eating disorders, among other areas. KT, a clinical psychologist and clinical lead of an inpatient, day hospital and outpatient ED program, reviewed the analysis and provided additional feedback. The implications of this data and our findings were impacted by TEK’s lived experience, as well as SPL research focus and KT’s professional experience. Since the time of data collection, TEK has joined a non-profit organization providing treatment to individuals with ED, and these experiences have also impacted how she interacts with and makes sense of the data.

### Data analysis

One of the proposed strengths of the Bardone-Cone definition is that it is transdiagnostic [19]. We suggest that any definition which is truly transdiagnostic should resonate with individuals across the illness spectrum and to this end, compared acceptance of the model across individuals with different diagnostic (i.e., history of anorexia nervosa vs. no history of anorexia nervosa) and symptom (i.e., self-reported binge eating, purging, or fasting in the past 4 weeks vs. no self-reported symptoms in the past 4 weeks) profiles. Although, given the person-centered nature of the research topic, we do not necessarily subscribe to assigning recovery categories, we felt that it was important to examine whether views on the defintion differed by whether individuals met the recovery criteria proposed by Bardone-Cone et al. [21]. As such, we also compared agreement with the model across individuals meeting full, partial, and no recovery criteria.

Data from the EDE-Q were used to classify participants according to their recovery status. Individuals were deemed to be in full recovery if their self-reported BMI was equal to or higher than 18.5 kg/m^2^, they reported no instances of self-reported binge eating, fasting, or purging (including self-induced vomiting, laxative misuse, and/or compensatory exercise) in the past 28 days,^2^ and EDE-Q Global and subscale scores less than one standard deviation above community norms (identified using Luce et al. [49] for women and Nagata et al. [50] for gender diverse individuals). Partial recovery was assigned if the individual met the weight and behavioural criteria but not the cognitive criterion. Of note, data used to determine recovery status were compiled from the original screening questionnaire which took place prior to the interview. Thus, there was a delay (typically between 1 and 3 weeks) between the collection of the data and interview data.

In order to determine individuals’ agreement with the Bardone-Cone definition qualitative content analysis was employed to quantify the number of participants who believed that the definition was appropriate. Based on the nature of their responses (i.e., we did not specifically ask about ‘agreement’ but inferred this from responses), each participant was categorized as either: (1) agreeing with the definition, (2) not agreeing with the definition, or (3) agreeing with parts of the definition. Criteria for agreement and examples of responses which were indicative of full, partial, and no agreement are included in Table 5. Lived experience perspectives on feasibility of the full recovery criteria (i.e., did participants feel it is possible to meet the criteria put forth) and sufficiency of the three domains (i.e., are the criteria which focus on weight, behaviours, and thoughts sufficient to capture recovery) were also computed at this stage and a list of elements identified as missing from the model was compiled (Table 6).

**Table 5:** Example excerpts demonstrating how agreement with the model was determined

	Criteria	Example quotations
Full recovery		
Agree	Individual uses language that suggests that they agree with the full recovery criteria (e.g., it fits) Individual does not use language indicating disagreement or ambivalence toward the definition	That definitely, that definitely like matches I would say it’s like pretty spot on kind of what I would imagine full recovery to be
Do not agree	Individual uses language indicating that they do not agree with the full recovery criteria (e.g., it does not fit)	Um so I think that definition is absolute bullshit
Agree with parts	Individual describes parts of the definition that they like and parts that they do not like	It makes sense as a textbook definition. Uh funny I didn’t realize they called it full recovery and partial. Um yeah it makes sense textbook, uh I think um living it though um and hearing full recovery without the definition is a bit deceiving Well in those terms, I think that the thoughts and behaviours are definitely more important than the actual weight
Partial recovery		
Agree	Individual uses language that suggests that they agree with the partial recovery criteria Individual does not use language indicating disagreement or ambivalence toward the definition	That’s a good term for like when I was like halfway through you know being weight restored or like you know as soon as you’re like weight restored but then there’s that like lag to catch up with the thinking
Do not agree	Individual uses language indicating that they do not agree with the partial recovery criteria	I don’t really think I like that term. It’s kind of like, I don’t know, you can’t- you can’t partially recover from like an ill- like an illness. Like you’re either recovering still or you’re recovered like there’s a 1 or 2 No that’s not good no. That, that is exactly that is a bit like using a BMI to judge someone, no I don’t like that
Agree with parts	Individual describes parts of the definition that they like and parts that they do not like	I suppose it, it makes sense. Although I feel like, it’s a bit ridiculous to expect someone to be in partial recovery and not to any behaviours whatsoever for 3 months. I feel like there would be aspects to that, that um I feel like if you’re still having those very intense thoughts, the behaviours would more than likely be in there?

**Table 6:** Proportion of individuals who felt that the model was appropriate

Views on Bardone-Cone et al. (2010a) model	Frequency (%)
*Agreement with full recovery criteria*	
Yes	8 (12.9)
Some are but some are not	35 (56.5)
No	19 (30.6)
*Feasibility of full recovery criteria*	
Yes	45 (72.6)
No	12 (19.4)
*Agreement with partial recovery criteria*	
Yes	14 (22.6)
Some are but some are not	26 (41. 9)
No	22 (35.5)
*Sufficiency of the three domains included (weight, behaviour, thoughts)*	
Yes	19 (30.6)
No	43 (69.4)
*If no, what should be added?*	
Moving away from eating disorder	3 (4.8)
Relationship with food	8 (12.9)
Relationship with activity/exercise	2 (3.2)
Daily living	11 (17.7)
Impact on other areas/quality of life	8 (12.9)
Social functioning	7 (11.3)
Emotional functioning	8 (12.9)
Self-perception and acceptance	5 (8.1)
Effective coping	3 (4.8)
More headspace	2 (3.2)
Identity outside of ED	3 (4.8)
Physical/biological functioning	3 (4.8)
Understanding of ED	2 (3.2)
Perception of recovery status	2 (3.2)

Percentages for whether full recovery criteria are possible do not add up to 100% because not all participants indicated this in their responses. The ‘what should be added’ categories were generated post-hoc based on participants qualitative responses

#### Quantitative analyses

To assess whether a history of anorexia nervosa diagnosis exclusively (versus multiple ED diagnoses or other ED diagnoses), current ED severity, and/or recovery status would impact participants’ views of the Bardone-Cone definition, Chi-Squared tests of independence and binary logistic regression analyses were computed using SPSS-28. Participants were categorized as agreeing with the full *and* partial recovery definitions or not. Chi-squared tests of independence were computed with agreement as the dependent variable and history of only anorexia nervosa diagnosis, presence of current ED symptoms, and recovery status as the independent variables, yielding three separate analyses. Binary logistic regression was computed with EDE-Q Global score (an indicator of ED psychopathology) as the predictor variable. A Bonferroni correction for multiple comparisons (n = 4) yielded a significance rate of alpha = 0.012.

#### Qualitative analyses

To better understand participants perspectives on the definition, codebook thematic analysis (TA) (e.g., [51, 52]) was employed. In contrast to more reflexive approaches to TA, codebook TA involves generating codes and themes early in the conceptualization process [53]. For the current study, relevant questions (in Table 4) and their responses were extracted to create the data set. This was then reviewed for the purpose of ensuring transcription accuracy (i.e., first author listened to the recordings while reading the transcripts) and then read and reread to increase familiarity. During this process, TEK kept handwritten notes in the margins. Notes were broad and referred to interview content and how this may relate to the research question. After compiling the first set of notes, TEK met with SPL to discuss initial observations. During this meeting, notes were reviewed, and possible codes were identified. After this, TEK reviewed the transcripts again and formalized codes. Codes referred to the single unit of information contained within the excerpt, as described in Braun and Clark [54]. These were then organized visually to construct preliminary themes. TEK chose to do this using post-it notes so that codes could be easily moved from one theme to another. TEK and SPL then met again to review the preliminary themes and possible subthemes. Themes and subthemes were finalized and named during this meeting. Any disagreements were resolved through discussion of the various points of view until the authors came to a consensus. TEK then reviewed transcripts again to select excerpts which illustrated the themes.

Throughout the analytic process, it seemed that concerns with Bardone-Cone’s definition of recovery were more dominant than the other two themes (i.e., understanding the need for a definition, agreement with the definition). We hypothesized that this may have, in part, been influenced by the nature of the questions. For instance, we asked whether the full or partial criteria fit for individuals. Participants who agreed with the definition tended not to elaborate on their responses leaving less text for analysis. Thus, we have tried to present themes with equal weight and highlight that for some people this model does fit, while also acknowledging the concerns that were identified with respect to Bardone-Cone’s definition of recovery.

## Results

### Lived experience perspectives on the proposed recovery criteria

The proportion of individuals who indicated agreement with the full and partial recovery criteria is listed in Table 6. The majority of participants expressed that some but not all aspects of the full (56.5%) and partial (41.9%) recovery criteria were appropriate, with about a third indicating that the criteria were not appropriate at all (30.6% and 35.5%, respectively). Only six participants (9.7%) provided responses suggesting that they agreed with both the full and partial criteria.

### Differential patterns of response

Chi-Squared Tests of Independence indicated that participants with a history of anorexia nervosa only, *X*^*2*^(1, n = 62) = 1.027, *p* = 0.331, *Cramer’s V* = 0.129, who did not report ED symptoms in the past four weeks, X^2^(1, n = 62) = 0.063, *p* = 0.802, *Cramer’s V* = 0.032, and who met full recovery status, X^2^(1, n = 62) = 1.896, *p* = 0.169, *Cramer’s V* = 0.175 were not more likely to report agreement with either the full or partial recovery definitions. Similarly, binary logistic regression revealed that there was no association between EDE-Q Global score and agreement with the full and partial recovery definitions, *ExpB* = 0.937 [0.533, 1.649], *p* = 0.882.

### Qualitative analysis

Participants’ responses broadly indicated three orientations toward the definitions: (1) understanding the need for a standard definition of recovery; (2) concerns with the definition; and (3) no concerns related to the definition. Within these, various subthemes emerged.

#### Understanding the need for a standard definition of recovery

A subset of participants indicated that they understood why researchers may seek to develop a consensus definition of recovery, as demonstrated in the following quotations:

‘I mean, as someone who does research and knows the term ‘operationalization’, I think there is value in defining it very strictly like that for the purposes of research, because you need something, like some tool to measure recovery’ (22-year-old woman).

‘I see the rationale behind it… like you need to put people in a place where like we feel okay enough for you to go out into the world so that people who are not as okay as you can come into this program’ (26-year-old woman).

Despite this understanding, participants suggested that the definition may have less utility in clinical settings, as illustrated by the following comment, ‘*I guess it’s helpful for research purposes I don’t know if it’s very clinically meaningful*’ (43-year-old woman). Another participant elaborated on this view, noting:

‘I think from the patient’s standpoint it’s a false sense of security, and it’s a really easy way to fall back into older behaviours or set yourself up to again be knocked down on your feet and not be prepared for how to deal with the outcome of that’ (26-year-old woman)

This quotation suggests that the use of such a definition in clinical settings may have iatrogenic implications in that individuals may view themselves to be more stable than they are and/or lack skills to manage the challenges that come with recovery. Alternatively, it was also noted that the model may overlook the diversity of recovery experiences:

‘I see its utility, but I don’t think it captures the diversity of experiences or the nuances that come with time periods of not feeling like you have eating disorder thoughts or weight fluctuations’ (22-year-old woman).

Thus, while participants could see *why* the definition may have been proposed, they expressed concerns related to its clinical utility.

#### Concerns with the definition

**Fig. 1:**
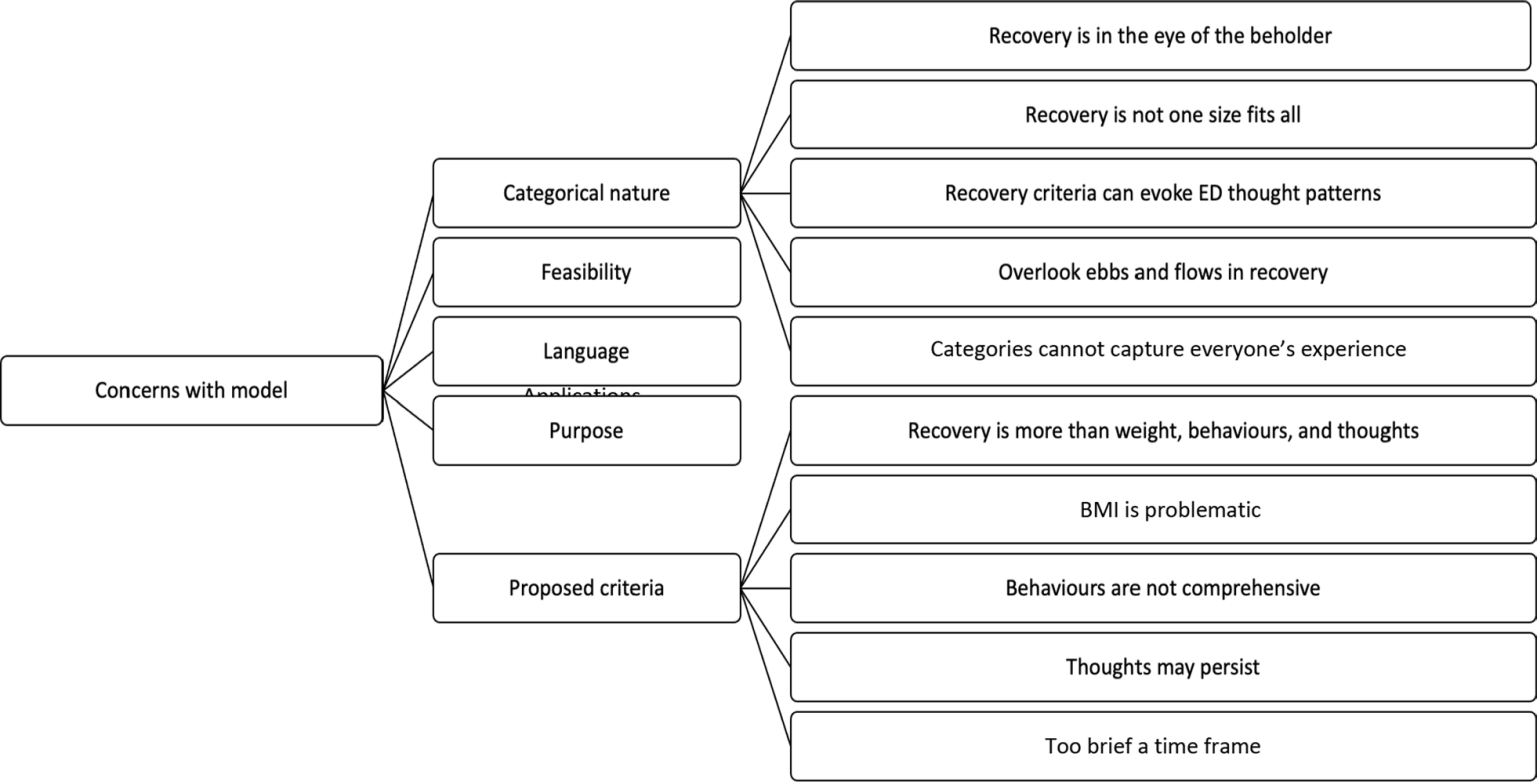
Thematic map of concerns identified by participants about the model proposed by Bardone-Cone et al. [21]

In line with the above, several participants expressed specific concerns across one or more of the following domains: (1) categorical nature of the definition; (2) proposed criteria; (3) feasibility of recovery definitions (i.e., can the criteria proposed be achieved); (4) use of ‘full’ and ‘partial’ language; (5) practical/real life applications. Within these domains, subthemes were identified and are presented in Fig. 1.

##### Categorical nature of the definition

Participants in the current study expressed concerns related to the categorical nature of the criteria proposed by Bardone-Cone et al. (2010a). One participant shared that ‘*I think it puts kind of that binary on it that isn’t conducive to actually feeling healthy and secure in your recovery*’ (24-year-old woman) and another described the model as ‘*black-and-white*’ (24-year-old woman). Concerns related to the categorical nature of the model generally fell into five sub-categories as described below.

*Recovery is in the eye of the beholder* Some participants described recovery as a self-defined phenomenon, as evidenced by the following: ‘*it’s also like to me, to each person individually who’s experiencing their recovery it’s kind of up to them to decide where they feel they are’* (25-year-old non-binary individual). These individuals expressed that categorizing people as being in full or partial recovery did not make sense given the subjective nature of recovery. For example, one participant noted:

*‘*I would be very cautious about calling that full recovery. I mean I would just call that recovery and then full recovery is something much deeper and much broader and something that you know any one individual can’t define for everyone. We all have to define it for ourselves and, and uh you know maybe some people don’t have a full recovery, I don’t know’ (33-year-old woman).

In this way, recovery is experienced as a self-constructed process that cannot be defined or prescribed by others.

*Recovery is not one-size-fits-all* In line with this, participants also described recovery as an individualized process, which cannot be circumscribed to two sets of criteria. For example, one individual remarked that, ‘*when we kind of like categorize things as like full or partial it’s back to that like dichotomous thinking that doesn’t leave room for like individual experience and individual um yeah just like ways of life*’ (24-year-old woman). Another participant provided a more tangible example noting:

‘the two girls that I’m still really good friends with, one would, would classify herself as being fully recovered for sure. Um I mean she said for her it’s like she never had an eating disorder and she’s been like that since um since we were in the hos- since we were discharged from the hospital essentially… And then my other friend um you know from her perspective she said it’s really only been in the last two years that she’s experienced um kind of that definition of, of recovery. So I think it’s possible but I think it looks different for everyone’ (26-year-old woman).

The example provided by this participant demonstrates how recovery trajectories can differ even when treatment experiences have been similar. Given this variability, participants suggest that a categorical means of defining recovery is not appropriate.

*Recovery criteria can evoke ED thinking patterns* An interesting point made by participants was the parallel between using recovery criteria and ED thought patterns. These individuals noted that elements such as perfectionism, which contribute to the development and maintenance of an ED, can also show up when looking at categories for recovery. Other participants noted that the recovery criteria categorize people into ‘*boxes*’ (26-year-old woman) and can evoke feelings of comparison (44-year-old woman). One individual noted:

‘it sounds like another goal to attain. Uh which I think is really unhealthy especially when it comes to eating disorders because our entire, well I shouldn’t say our, like my entire eating disorder experience was around reaching a goal weight. And so if there’s another goal that I have to reach that is literally unattainable like my ideal weight was, um it’s it’s not helpful at all I don’t think’ (23-year-old woman).

As is illustrated here, the use of recovery criteria may elicit perfectionist tendencies which can be characteristic of EDs. Indeed, some individuals suggested that hearing about these categories would have been detrimental to their recovery process: ‘*My eating disorder was very competitive, so I would have been like ‘it’s okay if I’m in partial recovery, but I can’t be in full recovery because then like I’m going to lose the eating disorder side of myself’’* (19-year-old woman). This again points to possible iatrogenic effects of presenting clients with a formal recovery model consisting of specific criteria and labels (i.e., it may pull for ED thought patterns such as perfectionism or black-and-white thinking).

*Overlooks ebbs and flows in recovery* One of the most frequent criticisms of the categorical nature of this model expressed by study participants was that it overlooks expected ebbs and flows in the recovery process. Individuals endorsing this subtheme noted that it would not be uncommon to have periods of greater ease and challenge as part of recovery. One participant, a 26-year-old woman, shared ‘*I’ve had three, four months where I’m doing awesome and I fall into that category, and then something happens in month 5, it’s a little bit too much for me to handle and months 6, 7, 8, 9, I’m back into full-blown symptoms.*’ This example illustrates the non-linear nature of recovery and how a single set of criteria at one-time point does not necessarily give the full picture. In line with this, some individuals suggested that ‘in recovery’ or ‘recovering’ would be a better way to categorize the process while accounting for natural variations throughout. For instance, one participant noted, ‘*I don’t know if I would really agree with discussing it as full or partial recovery, I just look at it as ‘in recovery’. During recovery then you have those struggles where you’re thinking or you’re using behaviours more, but then there are times where you’re not’* (19-year-old woman).

*Categories cannot capture everyone’s experience* Participants noted that the categorical nature of the proposed recovery criteria cannot capture everyone’s experiences, which means that “*inevitably [people] will fall through the cracks because you’re trying to create boxes”* [26-year-old woman]. Though similar to *One size does not fit all,* which emphasizes the diversity within people's recovery experiences*,* this theme is distinct in that it highlights how many individuals’ experiences will not be captured by these criteria. Participants expressed concern that some individuals would then be categorized as not being in recovery, when in fact they have made improvements in many domains. Similarly, some participants felt that categorizing participants as in full or partial recovery may minimize recovery efforts. For example, one individual shared how the presentation of these categories felt like an erasure of the hard work that she has done over the past several years:

*‘*both of those terms partial recovery or total recovery would both erase the achievements I’ve made. Like can we just focus on that for a moment like. That, the incredible achievements I’ve made that has taken my lifetime to get here they both erase that. They, they would not acknowledge that any progress has been made. And it’s taken my lifetime to get to this point of healing’ (28-year-old woman)

For people with lived experience who have worked incredibly hard on their recovery journeys, these limited and restricted criteria can therefore, feel minimizing of their efforts and progress.

##### Proposed criteria

In addition to concerns related to the categorical nature of these criteria, individuals in the current study expressed concerns with the specific criteria proposed. These broadly fell into five subcategories, which are described below.

*Recovery is so much more than weight, behaviours, and thoughts* Overwhelmingly, participants noted that recovery comprised more than just weight, behaviours, and thoughts. For example, one participant shared:

‘whenever I’ve heard people talk about being recovered it’s never being defined by any of those three things. It’s been like oh yeah I’ve gotten really in recovery the last few years, I’ve been travelling so much, and like I don’t, I can like drink alcohol now and that doesn’t bother me and I’m like not counting calories’ (23-year-old woman)

As it is described here, recovery seems more complex and multi-faceted than what is captured by the Bardone-Cone definition. This was also reflected by participants who noted ‘*I feel like that’s just a very surface level full recovery*’ (38-year-old woman) and ‘*I think it’s missing like the aspects of life that aren’t the eating disorder*’ (23-year-old woman). In this way, recovery is conceptualized by people with lived experience as being much broader and ‘*deeper*’ (33-year-old woman) than what is captured in the proposed criteria. Commensurate with these views, a list of the most common responses for what should be included in the context of recovery—beyond (or in place of) weight, behaviour, and thoughts—is listed in Table 6.

*BMI is problematic* Several participants in the current study expressed concerns related to the inclusion of BMI in recovery criteria. Here, they highlighted the flawed nature of this metric:

*‘*BMI is kind of like bullshit. It was invented by a mathematician not for the purposes intended, and the range of normal BMI is just arbitrary. Like there’s lots of people who would not be in a normal BMI and would be healthy, that’s their body’s optimal weight’ (25-year-old woman).

‘I’m a big opponent of BMI because you know it’s interesting the article in 1972 that established the use of BMI um actually didn’t- excluded women from the study. Um they didn’t even say BMI should be used for individuals they said it should be used from a population basis’ (29-year-old woman).

According to these individuals, the inclusion of BMI is problematic because of the flaws inherent in the construct of BMI. Other participants called attention to potential challenges related to natural weight diversity. Some expressed concern that individuals may have a BMI naturally lower than 18.5 (e.g., ‘*I’ve always even before the eating disorder been like underweight’* [34-year-old woman]) while others cited concerns that the vast majority of people with eating disorders never reach a BMI lower than 18.5 (e.g., ‘*the whole BMI being greater than 18 or whatever is kind of an issue in my opinion. Because obviously there can be people with severe eating disorders that aren’t below that to begin with’* [24-year-old woman]). As a solution to this, some participants recommended that weight not be the sole indicator, that physical measures be used, and/or that normalized eating and the absence of compensatory behaviours be considered instead.

*Behavioural criteria are not comprehensive* Some participants indicated concerns that the behaviours included in the proposed definition (i.e., fasting for 8+ h, binge eating, purging) were not sufficient to represent the breadth of ED behaviours relevant to recovery (e.g., ‘*I think it may not capture all of the eating disordered behaviours or symptoms that people might experience’* [25-year-old woman]). One participant provided an overview of some possible behaviours that could be overlooked:

‘there’s so many sneaky behaviours... I can have food rules but I’m not starving and I’m not bingeing and purging and things like that. Like I can still be really uncomfortable around food and make certain decisions that are based solely on my eating disorder, what makes my eating disorder brain happy and still get everything in…And maybe it’s things that I know regular people aren’t aware of like this has more calories than that but eating disorder people are. Like I can be like yes, I would prefer the vanilla instead of the chocolate and people are like it’s preference and I’m like no’ (24-year-old woman)

Taken together, from a lived experience perspective, there are other significant/important ED behaviours that have historically not been recognized and as such, the proposed definition may not capture the diversity of ED recovery experiences.

*ED-related thoughts may persist in recovery* A subset of individuals expressed concerns related to the ‘minimal thoughts’ criterion. While some felt that comparing ED psychopathology to a community sample accurately reflected the normative nature of diet culture thoughts (‘*It’s not that there’s never gonna be any thoughts anymore’* [21-year-old genderqueer individual]), most indicated that ED-related thoughts would likely be greater than in people who had never experienced an ED. One individual shared:

‘an eating disorder is something that you can recover from but the thoughts are gonna be a lifelong condition cause there’s a reason those thoughts happen in the first place and why they manifested’ (20-year-old woman).

Moreover, participants reported that it is the way that one responds to the thoughts (rather than the presence of thoughts themselves) that is indicative of recovery: ‘*Like I have eating disordered thoughts every day. It’s whether, it’s how I choose to hold them that I think is what defines being healthy’* (23-year-old woman). Thus, for individuals with lived experience there is a general acknowledgement that ED thoughts will persist in recovery (whether they are equivalent to the general population or higher) but that the more important question is how individuals are able to respond to and interact with those thoughts.

*Too brief a time frame* Finally, participants reported concerns related to the three-month timeframe used in the Bardone-Cone definition. Participants noted that compared to the time that they had had the ED, three months was insufficient to capture recovery:

‘You couldn’t consider somebody who’s had an eating disorder whether it’s binge, purge, anorexia who’s survived three months to say they have full recovery, bullshit no. I would never use that as a descriptive, three months is a drop in the ocean for someone with an eating disorder’ (63-year-old woman)

According to these participants, it would require a longer period of well-being in order to consider someone to be in full or partial recovery (‘*I think it’s like a more long-term’* [24-year-old woman]). Moreover, others highlighted that the physical effects of an ED alone will take significant time to heal, as evidenced by the following excerpt: ‘*I’m really not sure you can make that determination after *3* months. Especially if somebody’s recovering from being severely clinically malnourished. Um you know it’s something that’s gonna take a long time’* (26-year-old woman).

##### Feasibility of recovery definitions

Participants also reported concerns related to the feasibility of the proposed definitions, that is whether these criteria are achievable. Specifically, and similar to what was noted above for *thoughts may persist in recovery,* some participants reported feeling that the persistence of ED thoughts hindered full recovery. For example, one participant shared,

‘I don’t know if like you could ever just stop having those thoughts, like I don’t know what the research is on that. Because I feel like I’m in a better place after 5 months but I still do have those thoughts like seeing the weight or seeing myself in the mirror sometime it kind of just the automatic thought is still there that you know just probably just plays on a record reel’ (33-year-old woman).

Other participants reported that it would be impossible to have ED thoughts and not act on these—in this way partial recovery was deemed to be not plausible because there is no way to have thoughts without behaviours. Participants, therefore, noted concerns related to the feasibility of both full and partial recovery which may be tied to or distinct from the persistence of thoughts in recovery.

##### Use of ‘full’ and ‘partial’ language

Participants also expressed concerns related to the use of ‘full’ and ‘partial’ recovery language. Concerns were primarily related to the connotations of finality and partiality associated with the terms. Participants noted that saying ‘full recovery’ connoted a sense that something was finished. One participant, a 33-year-old woman, shared, ‘*what concerns me about that language is that it just seems so final, when it’s not something, that—it can’t be final*.’ Consistent with the idea that recovery involves ebbs and flows, participants seemed to feel that ‘full recovery’ did not necessarily capture the non-linear and ongoing nature of recovery. In terms of partial recovery, participants expressed feeling like their accomplishments had been minimized. One participant even expressed offense noting, ‘*it’s just so like minimizing the hard work that I do and the success that I do have and a bit offensive*’ (37-year-old woman). Another participant likened it to getting ‘*50% on your recovery test*’ (29-year-old woman), which may result in people feeling as though they have failed.

##### Practical/real life applications

Finally, some participants questioned the applications of the definition. Participants expressed tangible concerns about how the use of these definitions may impact individuals seeking services. One individual shared concern that treatment centers using the partial recovery criteria may prematurely discharge patients on the basis of weight alone which may lead to worse outcomes:

‘I’ve had several friends and acquaintances who come into the hospital, they bump their weight above the BMI and then they kick them out, and then they’re just like, now they’re even worse than they started because emotionally they’re even more broken because…you put a band-aid on the problem, but like, you kind of like, before they came in with like a scrape, and then instead of healing the scrape, you like stabbed their leg, took it out, and put a band-aid on it, and then pushed them out’ (26-year-old woman)

There were also concerns, particularly from participants residing in the United States, about how such criteria may impact insurance coverage, as indicated by the following: ‘*it’s like when they have these arbitrary cut offs, you know sometimes they use that as an excuse not to cover things’* (29-year-old woman).

#### No concerns related to the definition

These concerns notwithstanding, there was a subset of participants who felt that the definition is accurate and appropriate. Responses from these individuals typically fell into three subcategories described below.

##### Criteria are consistent with experience

Participants who endorsed the definition often did so because it was consistent with their lived experience. An individual who self-reported as being in full recovery noted, ‘*I would say like what I have now is no different than just the average person on the street. The dissatisfaction may be like a slight, slight bit more just because of the history*’ (41-year-old woman). Other participants noted that the partial recovery experience was consistent with their lived experience:

‘It definitely sort of does describe how I feel like I was like a period of time that I was in where I wasn’t exhibiting those behaviours but the thoughts were still really prevalent’ (25-year-old woman)

‘Cause I do think that like that for me was the, the most difficult thing like yes, I was hungry all the time but I also just the amount of brain space and obsessive thoughts that my eating disorder took up was the most difficult part of it and I think it was the last to dissipate also’ (24-year-old woman)

Thus, for some people the criteria proposed by Bardone-Cone and colleagues [20] seem to reflect their experience in recovery.

##### Partial recovery acknowledges progress and room for growth

When talking about this recovery model, participants also commented on how the partial recovery category was appropriate because it acknowledged that an individual had made some progress but still had a way to go. For example, a participant shared,

‘It kinda fits because I feel like that’s… I’ve done a lot of work, but I’m not there so I wouldn’t want to say I’m fully recovered. I wouldn’t want to say that I haven’t done anything, so I think it fits well with me. And I’ve seen where it could go, so I feel better at that… Yeah’ (38-year-old woman).

Another participant described how using the term partial could be validating for individuals who have made some progress on their journey: ‘*I think it’s, it’s good to kind of put in that context of uh partial because if you do have the thoughts you’re not gonna feel like well that was a waste, why did I do treatment if it didn’t cure me’* (33-year-old woman). For these individuals, the inclusion of a partial recovery category was validating in that it acknowledged both the progress and the need for continued growth.

##### Gives hope and direction

The final reason that participants provided in support of the definition was that talking about full recovery was aspirational and gave people hope. For some people, they expressed that it was important to talk about full recovery as a means of demonstrating that it was possible. One individual noted ‘*I think it’s appropriate cause the more we talk about it as a thing the more it’s like yeah that’s something you can do*’ (26-year-old woman), while another shared, ‘*I think it’s important to talk about full recovery to let people know that there is hope to recover from an eating disorder’* (27-year-old woman). For these individuals, talking about full recovery is a means of inspiring hope. Other participants shared that full recovery can also give you something to aim for: ‘*I think that would definitely be what you’d aim for in full recovery’* (59-year-old woman). Full recovery is then seen as an aspirational concept which can give people hope and direction.

## Discussion

The primary goal of the current study was to determine individuals’ with lived experience perspectives on a commonly cited definition of ED recovery [21], including whether they agreed with these definitions. A secondary aim was to examine whether agreement with the definition differed based on diagnostic history and/or current severity of symptoms. Findings indicated that most participants had concerns with some or all aspects of the proposed definition. Under ten percent of the sample agreed with both the full and partial definitions as written, and diagnostic history and current symptom severity did not predict likelihood of endorsing full and partial recovery definitions. Interestingly, participants’ recovery status according to the Bardone-Cone definition of recovery also did not predict agreement with the model, suggesting that meeting full recovery criteria does not increase likelihood of identifying with this framework. Looking at qualitative responses gave more insight into participants’ views on the model.

Participants expressed an understanding of *why* researchers and clinicians would strive for a standard definition of recovery, consistent with what has been reported by service users with anorexia nervosa [42]. Moreover, there was a subset of individuals who felt that the definitions were appropriate, because they fit with participants’ experience of recovery, accurately described the partial recovery state, and provided hope and direction for full recovery. It is inaccurate then, to say that all individuals have concerns with this model, and indeed, for some individuals full and partial recovery defined in this way may best describe their lived experience. This notwithstanding, there were many more individuals who expressed concerns related to the model.

First, participants reported concerns related to the *categorical nature* of the model. Individuals believed that recovery is too subjective (*Recovery is in the eye of the beholder*) and individualized (*Recovery is not one-size-fits-all*) to fit into boxes and that doing so ultimately results in individuals falling through the cracks (*Categories cannot capture everyone*). They also highlighted that recovery is a non-linear process (*Overlooks ebbs and flows*), which cannot be captured by discrete categories, consistent with a body of qualitative research which notes that individuals with lived experience described recovery as a process or journey that fluctuates over time (e.g., [39, 41, 42]). Notably, participants commented on possible iatrogenic effects of using a categorical model of recovery in that it may evoke perfectionism, competitiveness, or desire to be unwell among individuals with EDs (*Recovery criteria can evoke ED patterns of thinking*). Related to its categorical nature, participants explicitly noted concerns with the *language* used in this model. Specifically, they shared that the use of ‘full’ connoted a finality, while ‘partial’ suggested only a partial success.

Additionally, participants expressed concerns related to the *proposed recovery criteria*. First and foremost, participants reported that recovery cannot be defined by ED-related symptoms alone (*Recovery is more than weight, behaviours, and thoughts*). Consistent with past qualitative research, participants mentioned that recovery includes amelioration of ED symptoms but that these alone are not sufficient to capture the complexity of recovery (see [40] for qualitative meta-synthesis). Based on participants’ responses, it seems that from lived experience perspectives, recovery comprises not only the absence of symptoms but also the presence of positive areas of functioning, which has also been suggested by Streigel Weissman [55].

Consistent with past qualitative research, participants highlighted the importance of psychological well-being [39–41, 56, 57], improved coping [57–61], identity outside of the eating disorder [41, 56, 59, 62–66], self-perception and acceptance [41], and improved social functioning [39–41, 58, 60–62, 66–68]. Further, participants noted the importance of functioning in day-to-day life, what has been termed “functional recovery” in other models pertinent to mental illness [69], and improved relationship with food and exercise. Though some may argue that this latter point is captured by the EDE-Q, participants described the improved relationship as being deeper and more meaningful than is captured by the dietary restraint questions on the EDE-Q.

In addition to suggesting aspects be added to the definition, participants expressed concerns with the existing criteria. Specifically, and consistent with [42], participants expressed concerns related to the inclusion of BMI (*BMI is problematic*), the comprehensiveness of behaviours (*Behaviours are not comprehensive*), and the ‘normalized’ thoughts criterion (*Thoughts may persist*), as well as the time frame (*Too brief a time frame*). Relatedly, participants noted concerns about the *feasibility* of these definitions. Similar to concerns about ‘normalized’ thoughts, participant responses indicated that coping with or managing ED thoughts seemed to be more realistic for individuals with lived ED experiences, versus reducing thoughts to a ‘non-clinical’ level.

Finally, participants expressed concerns related to the *practical or real-life applications* of these definitions. While some individuals acknowledged the utility of these definitions in research contexts, they indicated that it would not be useful to employ these in clinical settings. Explicit concerns were noted with respect to treatment decision-making and insurance policies; participants expressed fear that a partial recovery definition would be used to gatekeep access to treatment and would reinforce weight-centric views of EDs given the inclusion of a weight criterion. Therefore, despite an understanding of why researchers may want to have such a common definition, participants expressed confusion about the purpose of these definitions in clinical settings.

Overall, participants reported concerns related to the use of standardized recovery definitions more broadly (*categorical nature, language, applications*), as well as the specific criteria outlined in by Bardone-Cone et al. [21] (*proposed criteria, feasibility*). Given that criteria across ED recovery definitions tend to vary in terms of their cut-offs for the three domains included in the current definition and their time-frame, these concerns are likely to apply across a variety of definitions derived within the field and are not necessarily specific to this model.

### Implications

The findings from the current study highlight the importance of integrating lived experience perspectives in ED recovery definitions, as has been argued elsewhere [16]. Participants expressed that there is a difference between the usefulness of recovery definitinons in research and in clinical practice, suggesting that a single consensus definition, as has been historically argued for [15], may not be the most appropriate approach. Instead, separate definitions, whose purpose is explicitly outlined, may fit better with lived ED experience. For example, a definition of recovery employed in research may be focused on categorizing individuals for the purpose of identifying predictors of a positive outcome defined as x, y, and z, while a clinical model of recovery may be more multi-faceted and person-centered for the purpose of promoting positive treatment and recovery experiences. Given similar recent calls to action (e.g., [16, 55]) future efforts may wish to focus on developing a flexible, person-centered understanding of recovery.

#### Research

Given participants’ mixed feelings about recovery when it is defined in this way and the shift toward recovery as a self-defined experience [70], we suggest that researchers focus on the absence of a diagnosis or ‘remission,’ as opposed to recovery per se. We argue that it is incumbent on researchers to explicitly note how they define or operationalize positive outcomes in order to shift the focus toward transparency in research and to allow individuals with lived experience to reclaim their understanding of recovery. Shifting the focus to indicators of progress, absence of diagnosis, or ‘remission’ may address many of the concerns raised by participants in the current study while still acknowledging the need for outcome monitoring in research.

#### Clinical

Our findings suggest that in clinical practice understanding of recovery must comprise more than just symptom reduction. This is consistent with recovery-oriented practice (e.g., Mental Health Commission of Canada [71]; World Health Organization [70]) and points to the importance of recovery-oriented policy. In contrast to traditional medical framings of recovery which focus on symptom reduction, recovery-oriented practice highlights the importance of promoting hope, supporting individuals as experts in their own experience, and transforming systems, including working to reduce social determinants of poor mental health [71, 72]. Although recovery-oriented guidelines have been adopted in several countries [35, 73], elements of this framing are consistent with current treatment for EDs [34], and narratives of ED recovery include recovery-oriented elements (e.g., hope, autonomy) [66], recovery as a construct continues to be defined in terms of the medical model.

In addition to the findings reported here, recent reports (e.g., [16, 55]) have highlighted that this medical framing is insufficient from the perspective of individuals with lived experience. Instead, these studies suggest the use of a more comprehensive definition of recovery which aligns with recovery-oriented practice. For example, a dimensional approach [69] which has been proposed for mental illness more broadly and which acknowledges that recovery exists across various domains, one of which is symptom remission, may have utility. A recent open forum paper argued that in the context of EDs, and drawing from lived experience perspectives, definitions of ED recovery may be improved by deemphasizing a single weight target, highlighting the non-linear nature of ED thoughts and behaviours, and including quality of life and social functioning [16].

In line with this, participants’ emphasis on recovery being a complex and multifaceted phenomenon suggests that a person-centered model, which centers each individual’s varied and often unique experiences, may be better suited in clinical settings. Wetzler et al. [66] conducted a deductive analysis of ED recovery accounts in which they applied existing person-centered models of mental health recovery to participant narratives and reported that person-centered models may be applicable for individuals with EDs as well. We go further to argue that an ED-specific person-centered model which incorporates research findings and lived experience perspectives, as has been proposed in other areas of mental health (e.g., non-suicidal self-injury [74]), may have utility in that it would include areas identified by individuals with lived experience as being important in the ED recovery process (e.g., relationship with food and exercise) but which may not be relevant in other areas of mental health. We suggest that such a model should include areas of possible growth or change in the context of EDs but which may be deemed to have more or less importance by individuals with lived experience. In this way, the model would be inherently flexible and allow for differences in recovery experiences.

As has been stated by Lewis and Hasking [74] a person-centered model of recovery involves ongoing conversations with clients/patients about what ‘recovery’ means to them in order to facilitate engaged and meaningful recovery. Through these conversations, practitioners may identify areas of importance to individuals living with an ED, which offers direction in terms of treatment planning and outcome monitoring. Moreover, these conversations may offer opportunities for fostering hope and autonomy, consistent with recovery-oriented practice (e.g., Canadian and Australian guidelines [71, 72]). The possible benefits of this notwithstanding, future research is necessary to determine how people accessing services and working with health-professionals view this approach.

### Limitations and future directions

To our knowledge, the current study represents one of the largest qualitative studies on formal definitions of ED recovery and the first to explicitly examine views on the Bardone-Cone definition of recovery [21]. This notwithstanding, there are limitations that must be acknowledged in the interpretation of these findings. First, the sample was largely white, almost exclusively women, and entirely from Western countries. While we may anticipate that individuals of different ethnic, racial, and gender backgrounds may similarly have concerns with the Bardone-Cone recovery model given previous research looking at EDs in marginalized groups (e.g., [75, 76]), we must also acknowledge that areas of importance to these individuals may not have emerged in the current study. Thus, future research examining recovery models is encouraged to engage individuals with more diverse backgrounds than those in the present study.

Further, the sample primarily comprised individuals with a formal diagnosis and who had received treatment. Individuals with this history are more likely to be exposed to ‘recovery’ as a construct. Prior exposure to a recovery definition may lead to stronger reactions (positive or negative) in light of past experiences. For example, some participants expressed strong aversive reactions to the weight criterion because they had previously experienced involuntary weight restoration. It is conceivable that individuals who have not experienced treatment may have differing views on recovery and how to define it. Indeed, previous research in our lab has found that individuals associate recovery with treatment [77]. Thus, there is a need for additional research with individuals with EDs who have not received treatment to better understand their views on recovery. Additionally, most of the sample had received a diagnosis of anorexia nervosa at one time in their life (74.2%). Given the dearth of recovery research examining individuals with different diagnostic profiles (e.g., binge-eating disorder), it will be important to have recovery research that engages individuals with diverse ED experiences. Acknowledging these limitations, the current findings nevertheless point to a general dissatisfaction with an existing dominant recovery model.

## Conclusion

There has been a recent emphasis in the ED field on developing a consensus recovery definition [15]. To date, however, only one study has examined how individuals with lived ED experience view these definitions. In this study, we extended this research by examining views of 62 individuals with a past or present ED on a commonly cited model of recovery [21]. Though a minority of participants viewed this model as appropriate, and some could understand why it had been developed, the majority of participants indicated concerns related to the use of a model more generally, as well as the specific criteria included in this model. Thus, it is suggested that a single recovery model may not have utility in the field. Future research may benefit from distinguishing between recovery as an individually-defined phenomenon and related constructs such as remission (i.e., loss of diagnosis or absence of clinical symptoms). Along these lines, in clinical practice, we suggest that the development of an ED-specific person-centered model which situates people’s myriad lived experiences at the forefront, may have merit.

## Data Availability

The datasets used and/or analysed during the current study are available from the corresponding author on reasonable request.

## Abbreviations

ED: Eating disorder
BMI: Body Mass Index
EDE: Eating disorder examination
EDE-Q: Eating Disorder Examination—Questionnaire
OSFED: Other specified feeding or eating disorder
EDNOS: Eating disorder not otherwise specified
DSM: Diagnostics and statistical manual of mental disorders

## Footnotes

1 Other questions asked during the interview were part of another study and did not ask about the Bardone-Cone definition.

2 This criterion was altered from the original definition which specified 3 months [21] because of the timeframe used for the EDE-Q. Although we had data for three months from the EDE, the two measures were administered at different time points. Thus, we opted to use EDE-Q data for both the cognitive and behavioural criteria. In order to account for the shorter time frame, we used a slightly more conservative definition of behavioural abstinence which included compensatory exercise in addition to fasting, binge eating, and purging (self-induced vomiting or laxative misuse).
